# Case Report of a Triplet Pregnancy with Complete Hydatidiform Mole and Coexisting Twins

**DOI:** 10.1155/2022/2865342

**Published:** 2022-08-04

**Authors:** Marjorie H. Thompson, Nathaniel R. Miller, Shelby L. Haugan, Michael C. Gordon

**Affiliations:** ^1^University of Washington School of Medicine, USA; ^2^Providence Sacred Heart Maternal and Fetal Medicine, USA; ^3^Billings Clinic Bozeman, USA; ^4^Billings Clinic Maternal Fetal Medicine, USA

## Abstract

**Background:**

Triplet pregnancy with complete hydatidiform mole and coexisting twin fetuses is extremely rare with an unknown incidence.

**Case:**

Here, we present a case report of a pregnancy with twin fetuses and concurrent hydatidiform mole that resulted in the preterm delivery of one viable baby, the unfortunate intrauterine demise of the other twin, and successful treatment of gestational trophoblastic neoplasia in the postpartum period.

**Conclusion:**

This case highlights several important questions that arise for women who choose to carry a multiple gestation pregnancy with complete hydatidiform mole and describes complications that can occur. It is imperative to accurately assess risks and counsel individuals who elect to carry these pregnancies to provide the best possible outcomes.

## 1. Introduction

Twin pregnancy with complete hydatidiform mole and concurrent fetus is rare with an estimated occurrence of 1 in every 22,000 to 100,000 pregnancies [1–5]. The incidence of triplet pregnancies with twin fetuses and concurrent hydatidiform mole is unknown due to rarity of case reports.

Multiple gestation with a complete mole and coexisting fetus (CHMCF) is complicated by increased risk in spontaneous abortion, intrauterine fetal demise, preeclampsia, preterm labor, and hyperthyroidism. The chance of delivering a viable baby appears to be less than 50% according to current data [1, 6, 7]. Risk of progression of the mole to gestational trophoblastic neoplasia (GTN) does not appear to be increased for individuals who choose to carry CHMCF pregnancies to birth; however, it is considered somewhat controversial in the limited literature on this subject [5, 6]. Additionally, gestational age at molar evacuation or early termination of pregnancy does not appear to influence rate of progression to GTN [1, 8].

Here, we present a case report of a pregnancy with twin fetuses and concurrent hydatidiform mole that resulted in the delivery of one viable baby, the unfortunate intrauterine demise of the other twin, and successful treatment of gestational trophoblastic neoplasia in the postpartum period.

## 2. Case Presentation

**Figure 1:**
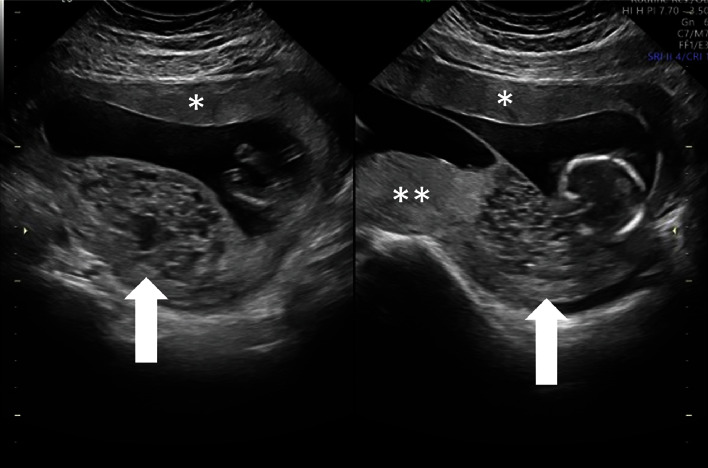
Ultrasound at 21 weeks showing three separate placentas and a dividing membrane ∗Twin A placenta. ∗∗Twin B placenta. Up arrow: molar placenta.

**Figure 2:**
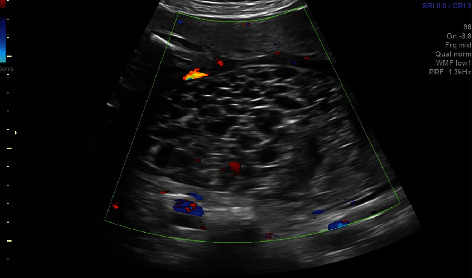
Close of color Doppler of molar placenta.

A 32-year-old healthy nulliparous woman achieved a diamniotic-dichorionic twin pregnancy with one cycle of clomiphene and timed intrauterine insemination. Her initial ultrasound was done at 7 weeks, and this exam noted 2 fetuses with an irregular third sac with no yolk sac or fetal pole and was thought to be a triplet pregnancy that was triamniotic-trichorionic. An ultrasound at 14 weeks noted a diamniotic-dichorionic twin with a posterior mass that was thought to be a degenerating fibroid. An 18-week ultrasound showed normal twin growth and anatomy, but a posterior uterine vascular mass was seen with a differential diagnosis of a fibroid versus a chorioangioma versus other multicystic uterine lesions. She presented for a comprehensive anatomic ultrasound at 21 weeks gestation with maternal fetal medicine to better evaluate this mass. This ultrasound revealed an abnormal third placenta along the posterior uterine wall with large multicystic structures, consistent with diamniotic-dichorionic twin pregnancy with coexisting complete hydatidiform mole (Figures 1 and 2). The abnormal third placenta was separated from the two normal twin placentas. The fetal anatomy was normal on both twins, and they were both appropriately grown. The diagnosis was confirmed by a second maternal-fetal medicine physician and a gynecologic-oncologist was also consulted. The B-human chorionic gonadotropin (B-hCG) was measured to be 276,568 mIU/mL. The chest X-ray was unremarkable. She was experiencing some nausea and fatigue but was otherwise healthy. Risks and benefits were discussed with her. Additionally, the option of pregnancy termination was offered given her increased risk for pregnancy complications and risk of developing persistent GTN. She elected to proceed with the pregnancy.

She was seen weekly by maternal-fetal medicine and had fetal growth ultrasounds obtained every 3-4 weeks. She took a daily 81 mg aspirin and performed home blood pressure readings to monitor for signs of early pre-eclampsia. The pregnancy proceeded without complication until 25 weeks.

At 25 weeks and 4 days gestation, she presented to the hospital for vaginal bleeding and was diagnosed with preterm premature rupture of membranes (PPROM) of twin A. She was given betamethasone, magnesium sulfate, and latency antibiotics. Her blood pressure readings were within normal limits, and a 24-hour urine protein was less than 300 mg. She had a low thyroid stimulating hormone (TSH); however, free thyroxine was within the upper limits of normal. A fetal growth scan was performed and was normal for both twins. The hydatidiform mole once again appeared to be separate and measured 19 cm in its largest diameter.

At 27 weeks and 3 days gestation, she had a transient increase in contractions in the early morning, and twin B was found to have a nonreactive nonstress test (NST). A biophysical profile (BPP) for both babies was 6/8 (− 2 due to decreased amniotic fluid in twin A and − 2 due to diminished breathing for twin B). Due to the overall BPP of 6/10 for twin B later that day, a repeat BPP was performed and revealed a demise of twin B. Over the next two days, the tocometer showed continued contractions and the NSTs were reactive for twin A.

At 27 weeks and 6 days gestation, due to vaginal bleeding, increased contractions, and cervical change, a placental abruption was suspected. Twin A was subsequently delivered by Cesarean delivery and weighed 2 lb 6 oz (1080 gm) with APGAR scores of 7/8, and there was meconium-stained amniotic fluid, but no obvious placental abruption was found. The suspected molar tissue was extracted completely without difficulty. Pathology confirmed the diagnosis that the third placenta was a complete mole. At autopsy, twin B weighed 2 lb 7 oz (1100 gm), and no gross developmental abnormalities or signs of infection were noted, and the placentas of the twins were unremarkable and confirmed diamniotic placentation.

One day after delivery, serum B-hCG measured 52,243.6 mIU/mL. The B-hCG initially decreased to 8,088.1 mIU/mL two weeks after delivery, but then increased to a peak of 17,562.8 mIU/mL three weeks after delivery. Due to this increase, she was diagnosed with GTN and was treated with intramuscular methotrexate: 30 mg for five consecutive days each week for one month. She desired to preserve fertility and thus hysterectomy was not recommended.

Her GTN responded to methotrexate. B-hCG lowered to 5mIU/mL, and she was treated with one more month of methotrexate and followed with weekly B-hCG. B-hCG was then followed every month for 12 months. She has remained on oral progesterone contraception during this time. One year after delivery, her B-hCG is still negative, and the surviving twin is doing well without any severe complications of prematurity.

## 3. Discussion

The initial diagnosis of a triplet pregnancy with complete hydatidiform mole and coexisting twin fetuses was challenging because of the rarity of the diagnosis. First trimester diagnosis is possible but appears to be more challenging, and the molar placenta can be incorrectly diagnosed as a subchorionic hematoma or an early miscarriage [9]. When the abnormal mole placenta is seen, the differential diagnosis typically includes a subchorionic hematoma, placental mesenchymal dysplasia, placental chorioangioma, an incomplete molar pregnancy, or triploidy. The important sonographic diagnostic features needed to make the correct diagnosis include seeing a separate atypical placenta with a Swiss cheese or cluster of grape appearance with otherwise normal fetal anatomy of the two intrauterine fetuses [9–11]. In addition, unlike a subchorionic hematoma, the molar placenta enlarges and has blood flow. The diagnosis is made easier with the finding of high B-hCG levels as was found in the case described.

Carrying a complete mole with coexisting fetus (CHMCF) pregnancy appears to have a significant increased risk of adverse perinatal outcomes, particularly a decreased chance of a viable pregnancy and increased risk for prematurity, particularly when compared with twin and triplet pregnancies without coexisting mole. In non-terminated singleton pregnancies with coexisting mole, the chances of a live birth are estimated to be 21-45% [1, 5, 6, 9, 10, 12]. This case study is the fourteenth (of which the authors are aware) to present a triplet pregnancy with twin fetuses and concurrent mole, for which 4 live fetuses have survived the neonatal period (29% live birth rate) [13].

There is also a high rate of preterm delivery in CHMCF. The mean gestational age at delivery for pregnancies that advance beyond 24 weeks gestation is 33.5 weeks gestation [5, 10, 11, 14]. The individual in this case study delivered at 27.9 weeks. Conversely, the average gestation for twin and triplet gestations without concurrent mole are 36 weeks and 32 weeks, respectively.

Much of the latest research on CHMCF has focused on risk of molar progression to GTN. However, these studies have also reported the incidence of obstetric complications in CHMCF. Vaginal bleeding is common, with an incidence as high as 76% in one literature review [14]. Risk of hyperthyroidism appears to be much lower, at about 15% estimated incidence [12]. In this case study, vaginal bleeding occurred later in her pregnancy, when she presented to the hospital at 25 weeks gestation and ultimately was diagnosed with a placental abruption. She did not have hyperthyroidism. Although the exact etiology of the cause of the stillbirth on twin B is uncertain, we speculate that it was due to a placental abruption.

The risk of preeclampsia is somewhat controversial. According to one cohort study of 77 CHMCF pregnancies performed by Sebire et al., the incidence of severe preeclampsia was 6%, which is reportedly higher than severe preeclampsia risk in normal twin pregnancies, but similar to rates of singleton complete hydatidiform molar pregnancies [6]. However, according to two literature reviews of cohort studies performed by Sukasi et al. and Wee et al., the estimate of preeclampsia in CHMCF ranges from 20 to 27% [12, 14]. A separate retrospective survey estimated the rate of preeclampsia and/or massive bleeding events at 14% [3]. This discrepancy might be explained by differences in the definitions of preeclampsia used. For example, Sebire et al. only reported the incidence of severe preeclampsia.

Premature rupture of membranes (PROM) was reported in one CHMCF pregnancy out of the reviewed literature [1]. In the literature review of CHMCF with two or more viable fetuses conducted by Takagi et al., they reported on a total of 13 pregnancies, and there were no incidences of PROM [13]. In our case study, it is unclear why this pregnancy was complicated by PPROM, and it is possible that this complication may not be associated with CHMCF or CHMCF with two viable fetuses and may simply have been a complication due to the multifetal pregnancy. In addition, whether the possible abruption was due to the molar placenta or simply a consequence of a multifetal pregnancy with PPROM is unknown. Abruptions are more common with multifetal pregnancies and with PPROM.

CHMCF pregnancies that are carried to birth do not appear to have a significantly increased rate of progression to gestational trophoblastic neoplasia (GTN) or more severe GTN in comparison to pregnancies that are aborted or have early elective terminations [5, 6]. However, this is still thought to be somewhat controversial, with multiple authors recommending that women need to be counseled about the potential benefits of pregnancy termination. The estimated progression to GTN appears to be 20-36% [5, 6, 9, 12]. However, one retrospective review of 14 CHMCF pregnancies reported a 50% rate of progression to GTN [1]. This discrepancy may be due to differences in study size, selection bias, differences in disease classification, or indications for chemotherapy.

## 4. Conclusion

This case highlights several important questions that arise for women who choose to carry a multiple gestation pregnancy with complete hydatidiform mole. Unfortunately, this pregnancy was complicated by intrauterine fetal demise, vaginal bleeding, and preterm delivery. There was also progression of the complete mole to gestational trophoblastic neoplasia after delivery. It is imperative to accurately assess risks and counsel individuals who elect to carry these pregnancies to provide the best possible outcomes for these women and their children.

## Data Availability

No data is included.
